# Integrated Application of Low-Intensity Pulsed Ultrasound in Diagnosis and Treatment of Atrophied Skeletal Muscle Induced in Tail-Suspended Rats

**DOI:** 10.3390/ijms231810369

**Published:** 2022-09-08

**Authors:** Xuebing Yang, Pan Li, Jiying Lei, Yichen Feng, Liang Tang, Jianzhong Guo

**Affiliations:** 1 Shaanxi Key Laboratory of Ultrasonics, Shaanxi Normal University, Xi’an 710119, China; 2 Junior Middle Department, Shanxi Modern Bilingual School, Taiyuan 030031, China; 3 Institute of Sports Biology, Shaanxi Normal University, Xi’an 710119, China

**Keywords:** muscle atrophy, LIPUS, gastrocnemius force, entropy, angular second moment

## Abstract

Long-term exposure to microgravity leads to muscle atrophy, which is primarily characterized by a loss of muscle mass and strength and reduces one′s functional capability. A weightlessness-induced muscle atrophy model was established using the tail suspension test to evaluate the intervention or therapeutic effect of low-intensity pulsed ultrasound (LIPUS) on muscle atrophy. The rats were divided into five groups at random: the model group (B), the normal control group (NC), the sham-ultrasound control group (SUC), the LIPUS of 50 mW/cm^2^ radiation group (50 UR), and the LIPUS of 150 mW/cm^2^ radiation group (150 UR). Body weight, gastrocnemius weight, muscle force, and B-ultrasound images were used to evaluate muscle atrophy status. Results showed that the body weight, gastrocnemius weight, and image entropy of the tail suspension group were significantly lower than those of the control group (*p* < 0.01), confirming the presence of muscle atrophy. Although the results show that the muscle force and two weights of the rats stimulated by LIPUS are still much smaller than those of the NC group, they are significantly different from those of the pure tail suspension B group (*p* < 0.01). On day 14, the gastrocnemius forces of the rats exposed to 50 mW/cm^2^ and 150 mW/cm^2^ LIPUS were 150% and 165% of those in the B group. The gastrocnemius weights were both 135% of those in the B group. This suggests that ultrasound can, to a certain extent, prevent muscular atrophy.

## 1. Introduction

Prolonged exposure to microgravity leads to multi-system deconditioning, especially for skeletal muscle systems [1]. Research shows that long-term space life can impair functionality [2] and neuromuscular control [3] and will directly lead to a substantial loss of muscle strength, with the strength decline primarily reflected in the loss of muscle mass [4]. Excessive loss of muscle or bone mass represents a risk to astronauts’ health, including the risk of irreversible changes that weaken skeletal integrity [5], which can impair the ability to perform activities of daily living. Therefore, there is an urgent need in aerospace medicine to find a method to prevent or treat muscle atrophy. A variety of physical therapy methods can promote the recovery of atrophic muscle function. The most commonly used physical therapy methods include low–medium-frequency electrotherapy [6,7] and hyperthermia [8]. Electrical stimulation can promote the isometric contraction of atrophic muscles, improve blood circulation and the oxidation capacity of muscle tissue, and improve anabolism and energy supply, which helps to maintain normal muscle tension. While clinical studies demonstrate efficacy, electrical stimulation is invasive due to the implantation of electrodes, which poses significant risks to the patient, such as infection due to surgery and the potential for secondary surgeries to repair or remove electrodes [9,10]. Moreover, resistance and endurance exercise have been shown to attenuate the effects of disuse muscle atrophy. However, not all disuse atrophy conditions allow for exercise training [11]. These exercises require large devices that are unlikely to be of significant use in small-capsule space vehicles for Mars missions [12].

Low-intensity pulsed ultrasound (LIPUS), as a physical stimulus, has the advantages of being non-invasive, portable, and targeted. Research shows that LIPUS can promote cell proliferation and differentiation in fibroblasts, Schwann cells, and other cell types [13], inhibit bone loss in ovariectomized rats or induced by weightlessness [14,15], and accelerate the fracture repair process [15,16]. Muscle atrophy is often accompanied by disorders of protein metabolism in muscle tissue, the increased decomposition of muscle protein, and the thinning or even disappearance of muscle fibers [17,18]. Previous studies have shown that ultrasound can promote protein synthesis in tissues [19]. Based on the above, it can be inferred that LIPUS may be an effective method for the intervention and treatment of muscle atrophy.

In this study, we stimulated the hind limbs of tail-suspended rats using LIPUS (50 mW/cm^2^, 150 mW/cm^2^) and studied the intervention or therapeutic effect of LIPUS on muscle atrophy in terms of body weight, gastrocnemius weight, muscle force, and B-ultrasound images. The results show that LIPUS had a certain inhibitory effect on the loss of skeletal muscle atrophy.

## 2. Results

### 2.1. Model Validation and Muscle Force Changes

#### 2.1.1. Body Weight and the Gastrocnemius Weight

**Figure 1:**
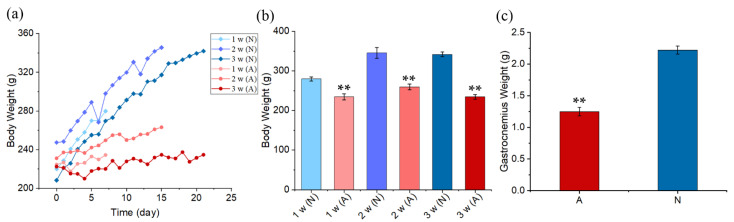
Body weight and gastrocnemius weight in a 21-day tail suspension test. (**a**) A 21-day continuous body weight change. (**b**) The body weights of the A group and the N group after 1 week, 2 weeks, and 3 weeks. (**c**) Gastrocnemius weights of the A group and the N group on day 21. The data are expressed as mean ± SE (*n* = 5 in each group), ** *p* < 0.01 vs. N group in the same week.

Body weight was measured every day during the experiment (Figure 1a), and those of the model group (A) and the control group (N) on the 7th day, 14th day, and 21st day were mainly analyzed and compared (Figure 1b). The body weight of the N group increased significantly in 21 days, and the weight of the A group increased slowly in the process of tail suspension and was significantly lower than that of the control group (*p* < 0.01). Gastrocnemius weight was measured on day 21, as shown in Figure 1c. The gastrocnemius weight in the A group was 56.2% of that in the N group, which showed the most significant difference (*p* < 0.01).

#### 2.1.2. Muscle Force

**Figure 2:**
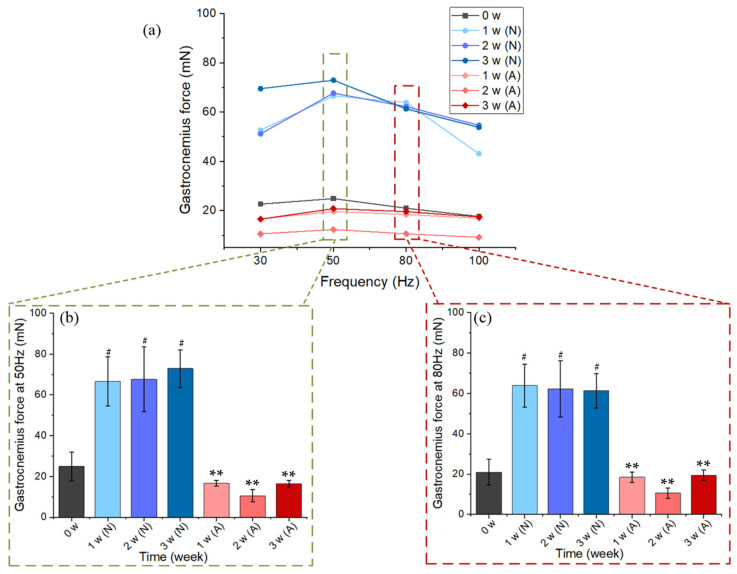
(**a**) The force–frequency relationship of 0–3 weeks. (**b**) The gastrocnemius force induced by LIPUS stimulation at 50 Hz. (**c**) The gastrocnemius force induced by LIPUS stimulation at 80 Hz. The data are expressed as mean ± SE (*n* = 5 in each group), ** *p* < 0.01 vs. N group in the same week, # *p* < 0.05 vs. 0 w.

Gastrocnemius muscle force was measured before the experiment (0 week, *n* = 5) and then was measured once a week during the experiment (after 1 week, 2 weeks, and 3 weeks, *n* = 5 for each group). The muscle was stimulated at increasing frequencies (30, 50, 80, and 100 Hz), and the force–frequency relationship was analyzed (Figure 2a). As can be seen from Figure 2a, the muscle forces were relatively large at 50 and 80 Hz. The muscle forces induced by 50 Hz and 80 Hz electric stimulation were analyzed between groups, respectively. From Figure 2b,c, the muscle force of rats in the A group decreased significantly, especially on day 14, and then increased slightly on day 21. This shows that the muscle had atrophied, which verifies the reliability of the model.

#### 2.1.3. Texture Analysis of B-Ultrasonic Images on Day 21

**Figure 3:**
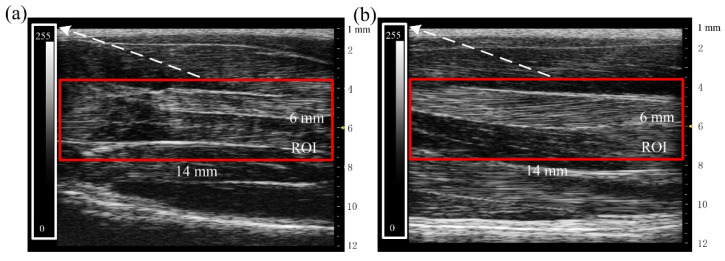
B-ultrasonic images of the rat hind limbs on day 21. (**a**) B-ultrasonic image of the A group. (**b**) B-ultrasonic image of the N group. In the figure, the red rectangle is the region of interest (ROI), the white rectangle is the scale bar of the ROI, the yellow arrow represents the focus depth of array transducer.

Figure 3a,b show the B-ultrasonic image of the hind limbs of rats in the A group and the N group, respectively, from which it can be found that the muscle thickness of tail-suspended rats (A) decreased, the myofilament trend was disordered, and the muscle filaments at some positions were fuzzy and difficult to distinguish. In the control group (N), the deep fascia of the muscle bundle was complete, the muscle bundle was clearly visible, and the structural morphology was normal.

The red rectangle in Figure 3 was used as the region of interest (ROI). The grayscale mean value, variance value, ASM, ENT, and contrast were identified from ROIs to quantitatively evaluate the state of muscle atrophy. The statistical results are shown in Table 1. Compared with the N group, the mean value, variance value, contrast, and ENT of the A group increased significantly, while the ASM decreased significantly (** *p* < 0.01).

### 2.2. Interventionary Study of LIPUS on Muscle Atrophy

#### 2.2.1. Body Weight and Gastrocnemius Weight

**Figure 4:**
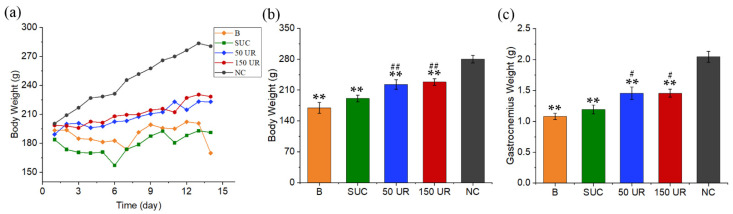
Body weight and gastrocnemius weight in a 14-day tail suspension test. (**a**) A 14-day continuous body weight change. (**b**) The body weights of the B, SUC, 50 UR, 150 UR, and NC groups on day 14. (**c**) Gastrocnemius weights of the B, SUC, 50 UR, 150 UR, and NC groups on day 14. The data are expressed as mean ± SE (*n* = 5 in each group), ** *p* < 0.01 vs. NC group, ## *p* < 0.01 vs. B group, # *p* < 0.05 vs. B group.

According to the previous experiment, we determined that the experimental period was 14 days. Body weights were measured every day during the experiment (Figure 4a). In contrast to the control group (NC), the weights of tail-suspended rats increased slowly. Gastrocnemius weight was measured on day 14. The two weights on day 14 were compared between the model group (B), sham-ultrasound control group (SUC), 50 mW/cm^2^ ultrasonic radiation group (50 UR), and 150 mW/cm^2^ ultrasonic radiation group (150 UR). Although the weights of the B, SUC, 50 UR, and 150 UR groups were significantly lower than those of the NC group, the body weights of rats stimulated by LIPUS (50 mW/cm^2^ and 150 mW/cm^2^) were 131% and 134% of those of the pure tail suspension group, as shown in Figure 4b. The gastrocnemius weights of the B, SUC, 50 UR, and 150 UR groups were 52.7%, 58.2%, 71.1%, and 71.1% of those of the NC group, respectively (Figure 4c). The gastrocnemius weights in the 50 UR and 150 UR were significantly related to those in the pure tail suspension group, which were 135% of those in the B group (*n* = 5 in each group, ** *p* < 0.01 vs. NC group, ## *p* < 0.01 vs. B group).

#### 2.2.2. Muscle Force

**Figure 5:**
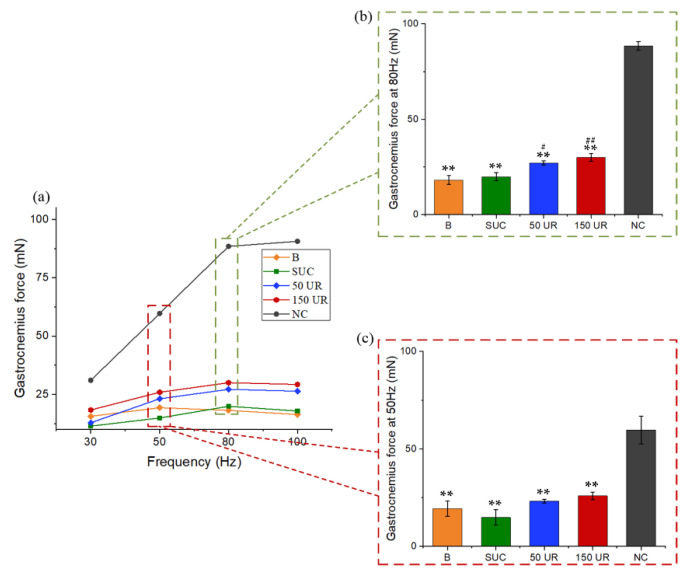
(**a**) The force–frequency relationship of five groups. (**b**) The gastrocnemius force induced by LIPUS stimulation at 50 Hz. (**c**) The gastrocnemius force induced by LIPUS stimulation at 80 Hz. The data are expressed as mean ± SE (*n* = 5 in each group). ** *p* < 0.01 vs. NC group, ## *p* < 0.01 vs. B group, # *p* < 0.05 vs. B group.

Gastrocnemius force was measured on day 14. Muscles were stimulated at different frequencies, namely 30, 50, 80, and 100 Hz, and the force–frequency relationships were obtained (Figure 5a). Due to muscle atrophy, the muscle force of the tail-suspended rats (B, SUC, 50 UR, and 150 UR) was significantly lower than that of the NC group at all frequencies. Similarly, the muscle forces induced by 50 Hz and 80 Hz electric stimulation were analyzed between groups, respectively. From Figure 5b, it can be seen that the trend of muscle force and weight between groups was completely consistent at 80 Hz. The gastrocnemius forces of the rats exposed to 50 mW/cm^2^ and 150 mW/cm^2^ LIPUS were significantly higher than those of the pure tail suspension group B, and the muscle force values were 150% and 165% of those in the B group at 80 Hz, and were 120% and 134% of those in the B group at 50 Hz, respectively (*n* = 5 in each group, ** *p* < 0.01 vs. NC, ## *p* < 0.01 vs. B group). It is proven that LIPUS has a certain role in the treatment of muscle atrophy.

#### 2.2.3. Texture Analysis of B-Ultrasonic Images on Day 14

**Figure 6:**
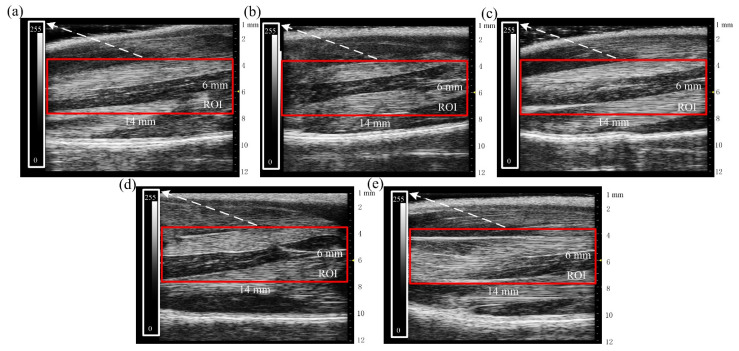
B-ultrasonic images of rats’ hind limbs in five groups on day 14. (**a**) B-ultrasonic image of the B group. (**b**) B-ultrasonic image of the SUC group. (**c**) B-ultrasonic image of the 50 UR group. (**d**) B-ultrasonic image of the 150 UR group. (**e**) B-ultrasonic image of the NC group. In the figure, the red rectangle is the region of interest (ROI), the white rectangle is the scale bar of the ROI, the yellow arrow represents the focus depth of array transducer.

At the end of the experiment, the B-ultrasonic images of the five groups (N, SUC, 50 UR, 150 UR) were collected (Figure 6). Figure 6a–d show B-ultrasonic images of tail-suspended rats. It is obvious that the muscle thicknesses of tail-suspended rats were smaller than those of the control group (NC) after 14 days, and the muscle filaments at some positions were blurred or even disappeared. The echo intensity was uneven. In the NC group, the muscle filaments were arranged in an orderly manner, and the morphology and structure of muscle bundles were complete and clear.

The red rectangle in Figure 6 was used as the region of interest (ROI). The grayscale mean value, variance value, ASM, ENT, and contrast were identified from ROIs to quantitatively evaluate the state of muscle atrophy. The statistical results are shown in Table 2. Compared with the NC group, the mean value, variance value, contrast, and ENT of the tail suspension group (B, SUC, 50 UR, and 150 UR) increased significantly, while the ASM decreased significantly (** *p* < 0.01 vs. NC group).

## 3. Discussion

Under long-term weightlessness or simulated weightlessness, disuse atrophy occurs in human or animal muscle tissue, especially in the lower-limb anti-gravity muscles. Relevant studies have shown that muscle atrophy will lead to a significant reduction in muscle volume, cross-sectional area, muscle force, body weight, and wet weight. A previous study showed that rats lost 5% of body weight and 19–24% of muscle weight (quadriceps, gastrocnemius, biceps, tibialis) after 17 days in a weightless environment [20]. Evidence exists that skeletal muscle mass and strength are reduced after as little as 7 days of space flight [21] or bed rest [22] and that they continue to decline with the length of exposure [23]. After 35 days of 6° head-down tilt bed rest, the muscle thickness decreased by 13–17% in the biceps femoris, vastus medialis obliquus, and vastus medialis longus [24]. Research suggests that quadricep disuse atrophy may contribute to quadricep weakness [25], which can lead to an approximately 7% deficit in total quadricep volume [26]. Skeletal muscle atrophy can damage many aspects of human health and quality of life. It is crucial in biomedicine and aerospace medicine to prevent or treat muscle atrophy. LIPUS has been widely used in clinical treatment and fundamental research. It is of great significance to the physiological regulation of the skeletal muscle system. To investigate whether LIPUS can prevent or treat weightless muscle atrophy, we built a muscle atrophy model via the tail suspension test and applied ultrasonic stimulation of different intensities (0 mW/cm^2^, 50 mW/cm^2^, and 150 mW/cm^2^) to the hind limbs of rats for 20 min every day. The state of muscle atrophy and the effect of ultrasound intervention were evaluated through body weight, gastrocnemius force, and B-ultrasound images.

### 3.1. Model Validation and Muscle Force Changes

A 21-day tail suspension experiment was carried out to verify the reliability of muscle atrophy induced by the tail suspension model. After suspension for one week, two weeks, and three weeks, the body weights of the model group (A) were 83.78%, 75.25%, and 68.66% of those in the control group (N), respectively (Figure 1b). The gastrocnemius muscle weight of the A group was also significantly lower than that of the N group (*p* < 0.01). In the second week, the muscle force of the A group declined further, and increased by 7.11% (50 Hz) and 14.64% (80 Hz) in the third week (Figure 2). As shown in the B-ultrasonic image in Figure 3, the muscle filament was disordered, and some muscle filaments disappeared. The above results confirm that muscle atrophy occurred, and the atrophying effect at 14 days was better. The results confirm and extend the characterization of the loss of muscle.

### 3.2. Interventionary Study of LIPUS on Muscle Atrophy

As a portable and non-invasive means, LIPUS has been widely used in various clinical medical research studies, and the medical use of ultrasound has been extended beyond imaging and diagnosis toward therapeutic applications [27]. Previous studies have shown that LIPUS can promote cell proliferation and differentiation [13], biosynthesis and metabolism [19], and the recovery of muscle injury. The sound intensity parameter widely used in previous studies is 30 mW/cm^2^. Sun et al. studied the effect of LIPUS on improving muscle atrophy by applying 30 mW/cm^2^ and 80 mW/cm^2^ of LIPUS. Their results showed that LIPUS had a tendency to prevent muscle strength loss, but there was no significant difference [28]. We used sound intensities of 50 mW/cm^2^ and 150 mW/cm^2^ here to stimulate muscles. On day 14 of the experiment, the body weight, gastrocnemius weight, and muscle force of the hind limbs of rats decreased significantly. The results showed that LIPUS had a certain effect by preventing muscle strength loss and weight loss. Although the muscle strength of the rats stimulated by LIPUS (50 mW/cm^2^, 150 mW/cm^2^) was still much smaller than that of the NC group, it was significantly different from that of the pure tail suspension B group (*p* < 0.01). Compared with the B group, the body weight and gastrocnemius weight of the 50 UR group increased by 31% and 35%, respectively; the gastrocnemius forces at 50 Hz increased by 20%, and those at 80 Hz increased by 50%; the body weight and gastrocnemius weight of the 150 UR group increased by 34% and 35%, respectively; the gastrocnemius forces at 50 Hz increased by 34%, and those at 80 Hz increased by 65%. The effects of the two sound intensities (50 and 150 mW/cm^2^) on preventing the loss of tissue mass and body weight were similar. There were significant differences between the two LIPUS groups and the B group, but no significant differences between the 50 UR group and the 150 UR group. It seems that LIPUS has a better effect in preventing gastrocnemius loss as the intensity of the ultrasound increases.

### 3.3. Texture Analysis of B-Ultrasonic Images

Non-invasive quantitative evaluation of microarchitectural changes is critical for understanding the pathophysiological mechanisms and evaluating their curative effects on skeletal muscle atrophy [29]. Several non-invasive imaging techniques have been used to assess pathological changes, such as magnetic resonance imaging (MRI) [30], computed tomography (CT) [31], dual-energy X-ray absorptiometry (DXA) [32], and ultrasonography [33]. Upon comprehensive comparison between these techniques, ultrasonography remains a low-cost, non-radiation, easily accessible technology. Biological tissue is mainly composed of water and fat, with only a small difference in acoustic impedance characteristics, which can transmit sound waves well [34]. When sound waves pass through tissues and encounter other tissues with different acoustical properties, such as muscle and fascia, sound waves will partially be reflected [35,36]. The ultrasound image is created based on these returning echoes and their temporal and acoustic properties [36]. The amount of returning echo per area determines the gray value of the image—that is, the echo intensity. Normal muscle is a hypoechoic structure in the ultrasonic image, and the appearance is black. Intramuscular fibrosis and fat infiltration usually occur after muscle atrophy, which will lead to more reflective interfaces within the tissue and give the muscle a whiter appearance [34]. Quantification of muscle echo intensity can be achieved with the grayscale statistic mean value. Previous studies reported that echo intensity increased with age-related reductions in muscle mass [37,38]. After 14 days of the tail suspension test, the grayscale mean value of the B-ultrasonic images in each group was as shown in Table 2. It can be found that the echo intensity after tail suspension increases significantly, and the echo intensity values of the 50 UR and 150 UR are between the normal control group and the pure tail suspension group. The large gray variance value and contrast of the tail suspension group also indicate that the echo intensity distribution is inhomogeneous. Quantification of structural changes in tissue can be achieved with the angular second moment (ASM) and entropy (ENT). ASM can describe the uniformity and regularity of the image texture, and ENT can be used to measure the disorder or complexity within an image [36]. Healthy skeletal muscle is largely homogenous in texture, whilst pathological muscles are often accompanied by intramuscular fibrosis and fat infiltration, so they have a greater degree of heterogeneity [39,40]. Muscle damage will lead to muscle texture disorder, and its image structure is more complex. Watanabe et al. [41] observed decreased ASM in sonograms of aging quadricep femoris muscle. Mahmoud-Ghoneim et al. [42] reported that ASM and ENT could discriminate between muscle conditions of normal, atrophy, and regeneration in rats. Martins-Bach [43] et al. suggested that contrast and ENT could identify different texture distributions of leg muscle groups in various mouse models of muscular dystrophy. Liu et al. [29] demonstrated that contrast significantly increased while ASM decreased in a diabetic group. Our experimental results show that after tail suspension, the image entropy and contrast of muscle increase while ASM decreases, which are consistent with previous studies [29,41]. There was no significant difference in ASM and ENT values between the ultrasonic irradiation groups (50 UR and 150 UR) and the pure tail suspension group.

## 4. Materials and Methods

The experiment was completed in two steps. In the first step, thirty-five SD male rats were selected as the experimental objects, and the tail suspension test was carried out to verify the reliability of the model and determine the appropriate experimental cycle. In the second step, the tail suspension experiment was carried out on twenty-five SD male rats purchased for the second time to study the intervention or therapeutic effect of LIPUS on muscle atrophy.

### 4.1. Animal

Thirty-five male Sprague Dawley (SD) rats at the age of 7 weeks (210–240 g) and twenty-five male SD rats at the age of 5 weeks (180–210 g) were obtained from the Laboratory Animal Breeding and Research Center of Xi’an Jiaotong University (Xi’an, China) in two batches and were housed in a controlled room (24 ± 0.5 °C, 60 ± 5% humidity, and 12-h light/dark cycle). All experiments were conducted with the approval of the Animal Ethical Committee of Shaanxi Normal University and in accordance with the Guide for the Care and Use of Laboratory Animals published by the US National Institutes of Health (NIH Publication No. 8023, revised in 1978).

### 4.2. Animal Model

To determine the most cost-effective and practical interventions available, it is necessary to establish a model to simulate the impact of microgravity on Earth. The weightlessness-induced muscle atrophy model was constructed by a tail suspension test. The specific method referred to the Morey–Holton model [44]: the tails of rats were suspended with chains to lift their hind limbs off the ground, and the axis of the rat’s body was at an angle of approximately 30° to the ground. The forelimbs of rats could move freely to ensure normal food and water intake.

### 4.3. Animal Grouping and Methods

#### 4.3.1. Model Validation and Muscle Force Changes

Thirty-five male Sprague Dawley rats were randomly divided into two groups: the model group (A) and the control group (N). The tails of rats in the model group were suspended to induce atrophy, and the rats in the control group were raised normally on the ground for 21 days, during which the body weight was measured daily and the gastrocnemius muscle contractile properties were measured on the 0th, 7th, 14th, and 21st days, with five rats each time in each group. The B-ultrasonic images of the rat hind limbs were collected, and the gastrocnemius muscles were harvested and weighed on day 21. The muscle force and B-ultrasonic image measurement methods are described in Section 4.4 and Section 4.5.

#### 4.3.2. Interventionary Study of LIPUS on Muscle Atrophy

Twenty-five male Sprague Dawley rats were randomly divided into five groups: the model group (B), the normal control group (NC), the sham-ultrasound control group (SUC), the LIPUS of 50 mW/cm^2^ radiation group (50 UR), and the LIPUS of 150 mW/cm^2^ radiation group (150 UR). Among them, the rats in the NC group were raised normally on the ground, while the rats in the other groups (B, SUC, 50UR, 150UR) were treated with tail suspension for 14 days. The hind limbs of rats in the SUC, 50 UR, and 150 UR groups were exposed to LIPUS (1 MHz, 20% duty cycle, 0, 50, and 150 mW/cm^2^, respectively) for 20 min every day. The transducers in the SUC group were only attached to the hind limbs of rats and did not emit. In the experiment process, body weight was measured daily. On day 14, the B-ultrasonic images of the rat hind limbs were collected, and the gastrocnemius muscles were dissected and weighed. Finally, the contractile properties of the gastrocnemius muscle were measured. The methods are as described in Section 4.4 and Section 4.5.

### 4.4. Muscle Force Collection Method

Rats were anesthetized in the abdominal cavity and the gastrocnemius muscles were dissected and immediately placed in a bath containing a nutrient solution (see Table 3 for the composition), which was kept at approximately 30 °C, and a 95% O_2_/5% CO_2_ gas mixture was continuously dissolved into the solution. One end of the muscle was tied to a Dual-Mode Muscle Lever System (305C-LR, Aurora Scientific Inc., Aurora, Canada) and the other end onto the bracket inside the tissue bath using a 4.0-gauge silk suture. Muscles were placed at optimal length (Lo). Thereafter, measurements of force–frequency were initiated. The muscle was stimulated at increasing frequencies—typically 30, 50, 80, and 100 Hz—causing it to contract and produce tension, and the corresponding muscle tension values at different stimulation frequencies were recorded. All data were recorded and analyzed using commercial software (DMC and DMA, Aurora Scientific).

### 4.5. B-Ultrasonic Image Acquisition and Texture Analysis

At the end of the experiments, the B-ultrasonic images of the gastrocnemius muscle were collected by the Vevo 1100 system (Visual Sonics, Toronto, ON, Canada). The linear probe with a frequency of 40 MHz was placed on the hind limb of the anesthetized rat and moved slowly and gently. The evaluation depth was set to 12 mm. The left and right legs of each rat were scanned twice.

Texture analysis is an important means of ultrasonic tissue characterization. A mathematical method, gray-level co-occurrence matrix (GLCM), was used for statistical 2D texture analysis. The mean and variance of grayscale values, contrast, angular second moment (ASM), and entropy (ENT) were identified from B-ultrasonic images and used to evaluate the state of muscle atrophy.

### 4.6. Statistical Analysis

Data are expressed as mean ± standard error (SE) and were analyzed using a one-way analysis of variance and post hoc Tukey multiple comparisons. Body weight, gastrocnemius weight, and gastrocnemius force produced by 50 Hz and 80 Hz stimulation were compared between groups. A *p*-value < 0.05 was considered statistically significant, and a *p*-value < 0.01 was considered a very significant difference.

## 5. Conclusions

In this study, we established a tail suspension model to simulate a weightless environment and induce muscle atrophy. Through the 21-day tail suspension experiment, we evaluated the process of muscle atrophy and found that the muscle force is the smallest, and the atrophy is the most obvious, on day 14. Through the 14-day tail suspension experiment, we analyzed the intervention effect of LIPUS on muscle atrophy. Results showed that LIPUS of 50 mW/cm^2^ reduced the loss of body weight, gastrocnemius weight, and muscle force by 19.0%, 18.4%, and 10.2%, and LIPUS of 150 mW/cm^2^ reduced the loss of body weight, gastrocnemius weight, and muscle force by 20.9%, 18.4%, and 13.4%. The results show that LIPUS can prevent the loss of skeletal muscle mass and muscle force and intervene in muscle atrophy. In conclusion, LIPUS proved to be a useful maneuver as a countermeasure to disuse muscle atrophy.

## Figures and Tables

**Table 1:** Statistical analysis table of texture analysis on day 21.

	A	N
Mean	88.90 ± 3 **	63.62 ± 3
Contrast	44.05 ± 1 *	34.54 ± 2
Variance	2629.12 ± 141 *	2133.27 ± 149
ASM	0.0057 ± 0.0001 **	0.0079 ± 0.0004
ENT	7.58 ± 0.03 **	7.26 ± 0.05

* *p* < 0.05, ** *p* < 0.01.

**Table 2:** Statistical analysis table of texture analysis on day 14.

	B	SUC	50 UR	150 UR	NC
Mean	95.32 ± 3 **	99.84 ± 3 **	93.48 ± 1 **	89.34 ± 1 *	75.40 ± 5
Contrast	42.49 ± 2 **	43.17 ± 1 **	41.01 ± 1 **	41.82 ± 1 **	34.32 ± 1
Variance	2734.38 ± 120 **	2736.26 ± 72 **	2634.58 ± 101 *	2729.00 ± 92 **	2153.78 ± 59
ASM	0.0055 ± 0.0001 **	0.0053 ± 0.0001 **	0.0056 ± 0.0001 **	0.0056 ± 0.0001 **	0.0071 ± 0.0005
ENT	7.62 ± 0.02 **	7.65 ± 0.02 **	7.63 ± 0.02 **	7.61 ± 0.02 **	7.38 ± 0.05

* *p* < 0.05 vs. NC group, ** *p* < 0.01 vs. NC group.

**Table 3:** Composition content table of muscle tissue nutrient solution.

	Concentration g/L	Molar Concentration mmol/g
NaCl	8.006	137
KCl	0.373	5
MgSO_4_	0.1203	1
NaHCO_3_	2.016	24
CaCl_2_	0.222	2
Glucose	4.324	11
Pyruvate Sodium	0.12	1

## Data Availability

The data that support the findings of this study are available from the corresponding author upon reasonable request.
